# Artificial Teeth Mounted in a Base of Gutta Percha

**Published:** 1856-01

**Authors:** 


*Artificial Teeth Mounted in a Base of Gutta Percha.*—It will be recollected by most of the readers of the Journal that Dr. A. Hill, of Norwalk, Conn., instituted a series of experiments with a view of ascertaining the practicability of using gutta percha as a base for artificial teeth, and that his efforts were so far crowned with success that he was enabled to employ it very advantageously, in many cases, for temporary sets. Soon after this announcement was made to the profession, Mr. Truman, of England, published a small treatise on the use of this article for the same purpose, and for the use of which, we believe, he obtained a patent. This treatise was reprinted some three or four years ago in the Journal, and we publish, in the present No., a letter from Mr. Slayton, dentist, of Indiana, who has recently succeeded in producing a very beautiful preparation of gutta percha for this purpose. It is so tinted as to resemble quite closely the color of the gums. Artificial teeth may be very securely mounted in a base of this preparation, which for temporary purposes, we have no doubt, will answer a very excellent purpose. It is certainly more congenial to the mouth than a metallic plate, and if it were durable and not liable to become offensive, would undoubtedly be a desideratum in the prosthesis of the teeth. We saw one upper set of artificial teeth mounted in this preparation which had been worn some four or five months. These appeared to be still quite securely arranged, and we were assured they were easily kept clean by means of a common tooth brush, castile soap and water.

The practical value of this preparation will soon, we have no doubt, be ascertained, as Mr. Slayton has already instructed many members of the profession in the manner of using it, and at the same time supplying them with the article. We would be glad to ascertain the result of the experience of some of our friends, who are using it. We will gladly lay such information as may be furnished on the subject before our readers. The preparation in question is kept for sale by Messrs. Jones, White & McCurdy, of Philadelphia.

